# A brief checklist of modifiable dementia risk factors (RF12): associations with cognitive and affective measures in an Italian cohort

**DOI:** 10.3389/fnagi.2026.1862742

**Published:** 2026-07-21

**Authors:** Francesco Giaquinto, Sara Assecondi, Nicola Vanacore, Daniele Romano, Paola Angelelli

**Affiliations:** 1 Department of Experimental Medicine - Lab of Applied Psychology and Intervention, University of Salento, P.zza Tancredi, Lecce, Italy; 2 Department of Psychology and Cognitive Science - DiPSCo, University of Trento, Corso Bettini, Rovereto, TN, Italy; 3 Center for Mind/Brain Sciences - CIMeC, University of Trento, Corso Bettini, Rovereto, TN, Italy; 4 National Centre for Disease Prevention and Health Promotion, Italian National Institute of Health, Via Giano Della Bella, Rome, Italy; 5 Department of Psychology and Milan Center for Neuroscience (NeuroMi), University of Milano-Bicocca, P.zza dell’Ateneo Nuovo, Milano, Italy

**Keywords:** cognition, cognitive decline, dementia, depression, modifiable risk factors, prevention, preventive health

## Abstract

**Introduction:**

Low education, hearing loss, traumatic brain injury, hypertension, alcohol abuse, obesity, smoking, depression, social isolation, physical inactivity, air pollution, and diabetes are modifiable lifestyle and experiential factors associated with an increased risk of developing dementia. This study introduces the 12-item Risk Factor Questionnaire (RF12), a brief checklist designed to capture these modifiable dementia risk factors. We present a preliminary investigation of criterion-related, cross-sectional associations between RF12 scores and cognitive/affective measures in a community-dwelling Italian cohort, comparing three scoring methods: self-report, informant-report, and a weighted score.

**Methods:**

A sample of 563 community-dwelling, cognitively healthy adults (326 females; mean age = 61.79 ± 10.19 years, range = 27–91; years of education = 11.76 ± 4.60, range = 3–30) completed the RF12 alongside standard cognitive assessments (MMSE, MoCA, SATURN, FAB) and a depression scale (GDS). Hierarchical regression analyses were conducted to examine the proportion of variance in cognitive and affective outcomes explained by RF12 scores, controlling for age, sex, and education.

**Results:**

A moderate correlation was observed between self- and informant-reported RF12 scores (Spearman’s *ρ* = 0.408, *p* < 0.001). Hierarchical regression analyses indicated that each RF12 scoring method accounted for unique, albeit small, portions of variance in cognitive and affective outcomes (incremental Δ*R*^2^ ranging from 0.8 to 2.3%, even after controlling for age, sex, and education.

**Discussion:**

The RF12 captures relevant dementia-related risk factors in a low-burden format, although the proportion of variance explained is modest. The tool may be useful for research, epidemiological applications, and prevention efforts. Further longitudinal studies are needed to evaluate its predictive validity in clinical populations and its potential utility in primary care and public health settings.

## 1 Introduction

Approximately 50 million people currently live with dementia worldwide, and this number is expected to triple within the next 25 years (Nichols et al., 2022). Over the past decade, several epidemiological studies (Anstey et al., 2022) have highlighted the important role of modifiable risk factors—lifestyle- and experience-related characteristics that can influence the likelihood of developing dementia—across lifespan. To quantify these risks, several individual risk indices have been developed, including CAIDE (Exalto et al., 2013), LIBRA (Deckers et al., 2015), and ANU-ADRI (Anstey et al., 2013). While these tools provide valuable information, they are often complex and require detailed clinical, cognitive, biomedical, imaging, and genetic data, which can limit their practical use in large-scale or primary care settings (Anstey et al., 2022). The most recent Lancet Commission reports (Livingston et al., 2017; Livingston et al., 2020; Livingston et al., 2024) on dementia prevention indicate that 12 to 14 modifiable risk factors may account for 40–45% of dementia cases, emphasizing the potential public health impact of identifying and targeting these factors. Recently, a self-report questionnaire was developed from a comprehensive systematic review of evidence and validated to capture modifiable risk factors for dementia (CogDrisk; Anstey et al., 2022), also featuring a validated short-form version (51 items accounting for 11 risk factors) (Anstey et al., 2024). However, unlike these comprehensive risk algorithms or longer self-report questionnaires, the RF12 is specifically designed as a very brief checklist (17 items accounting for 12 risk factors) to be used as a first-step, rapid evaluation in primary care, or in large-scale epidemiological and exploratory studies. Furthermore, it is the first self-report questionnaire for the assessment of modifiable risk factors for dementia developed in Italy.

The 2020 Lancet Commission report (Livingston et al., 2020)—which guided the conception of this study—identified the following modifiable risk factors: low educational attainment (early life); hearing loss, traumatic brain injury, hypertension, high alcohol consumption, and obesity (midlife); and smoking, depression, social isolation, physical inactivity, exposure to air pollution, and diabetes (later life). The 2024 update (Livingston et al., 2024) added untreated vision loss and high LDL cholesterol to this list. Together, these factors reflect lifestyle habits and life experiences that can influence neurocognitive health across the lifespan.

Addressing these modifiable risk factors could potentially reduce the prevalence of dementia, highlighting the importance of preventive strategies at both individual and population levels. Understanding how these factors relate to cognitive outcomes is therefore critical (Saunders et al., 2023). In this context, risk assessment tools play a key role in identifying individuals with elevated modifiable risk profiles who may benefit from lifestyle interventions or further evaluation. Epidemiological evidence suggests that subtle cognitive vulnerabilities may be present in midlife, highlighting the importance of early identification of cognitive changes. This underlines the potential utility of tools that assess both cognitive performance and modifiable lifestyle factors, even in populations that are ostensibly healthy (Zhou and Ashford, 2019).

First-level assessment for suspected cognitive decline is often conducted in primary care settings, such as general practitioners’ offices (Ford et al., 2018). These assessments typically involve a clinical interview and, occasionally, standard cognitive screening tests [e.g., MMSE (Folstein et al., 1975); MoCA (Nasreddine et al., 2005); GPcog (Brodaty et al., 2004)]. Recently, digital tools have gained attention for their potential to support rapid and standardized assessments (e.g., SATURN; Bissig et al., 2020; Giaquinto et al., 2024). However, a growing focus on lifestyle factors has highlighted the need for initial assessments to incorporate not only cognitive performance but also modifiable risk factors that could inform prevention strategies (Anstey et al., 2022).

In the primary care, brief and easy-to-administer tools are advantageous for addressing time constraints and facilitating rapid risk stratification (Sabbagh et al., 2020). This underscores the need for concise, self-report assessment tools that are easy to interpret and can support healthcare professionals in identifying high-risk dementia profiles requiring referral for specialized evaluation. The RF12 was developed as a concise, self-report tool to assess 12 modifiable dementia risk factors identified by the Lancet Commission, with an optional informant-report version for situations where self-report may be unreliable. The binary scoring system inevitably reduces variability; however, it was chosen to maximize usability in primary care.

The aims of the study were (i) to examine preliminary cross-sectional, criterion-related associations between RF12 scores and cognitive/affective measures (MMSE, MoCA, SATURN, FAB, GDS), (ii) to evaluate the incremental contribution of the RF12 in explaining variance in these outcomes beyond age, sex, and education, and (iii) to examine convergence between self- (RF12-Self and RF12-Weighted) and informant-report (RF12-Informant) versions.

## 2 Methods

This study used a single sample, cross-sectional design. The methods section of the present article was drafted with reference to the Transparency of Methods scale (Zogmaister et al., 2024) to ensure clear and comprehensive reporting, enhancing transparency and facilitating replication of the study (Supplementary Materials S.1).

### 2.1 Participants

A total of 609 residents of Puglia, in Southern Italy, were contacted and invited to participate in the study. Recruitment was carried out through the distribution of flyers in general practitioners’ offices, pharmacies, volunteer associations, a university department, and via the researchers’ social networks. Participants were invited to undergo a first-level assessment of their cognitive health and chose to participate voluntarily, without receiving any compensation. Exclusion criteria were based on “Equivalent Scores” (ES; Capitani and Laiacona, 2017), calculated referring to previously published normative studies [MMSE (Carpinelli Mazzi et al., 2020), SATURN (Giaquinto et al., 2024), and MoCA (Santangelo et al., 2015)]. Individuals scoring an ES = 0 on at least one of the three cognitive tests were excluded; this score is defined as a performance falling below the non-parametric outer tolerance limit, indicating a pathological result that lies beyond the threshold where 95% of the normative population is estimated to perform with 95% confidence (Capitani and Laiacona, 2017). The final sample included in the analyses consists of 563 participants (326 females, mean age: 61.79 ± 10.19, range: 27–91; years of education: 11.76 ± 4.60, range: 3–30). All participants were native Italian speakers and had no prior neurological or psychiatric diagnoses. Additionally, the RF12-informant version was completed by informants for a sub-sample of 174 participants.

### 2.2 Materials

#### 2.2.1 Risk factors—12 items: patient and informant versions

The RF12 is a checklist specifically developed to assess the presence of modifiable risk factors associated with the development of dementia, as outlined by Livingston et al. (2020). The 12 core risk factors included in the scale are: (1) low educational attainment, (2) hearing loss, (3) traumatic brain injury, (4) hypertension, (5) alcohol abuse, (6) obesity, (7) smoking, (8) depression, (9) social isolation, (10) physical inactivity, (11) air pollution, (12) and diabetes. Each item is formulated reference to the quantitative and temporal anchor points described in (Livingston et al., 2020), reported with the full questionnaire in the Supplementary materials S.2–S.6. Responses are dichotomous (“yes” or “no”), indicating whether the individual has experienced each risk factor at any point in their life. The RF12 is available in two formats: one completed by the target individual (self-report), and the other by an informant or caregiver who lives with the person or is familiar with their lifestyle. Both the Italian original and English translation of the scale are provided in the Supplementary materials S.1–S.4. This study analyses three scoring methods. The RF12-Self score sums the modifiable risk factors reported by the individual, yielding a total score from 0 to 12. The RF12-Informant score is calculated similarly based on the informant’s responses. The RF12-Weighted score is calculated as the sum of the weights assigned to each present risk factor: RF12-Weighted = ∑(wi × xi), where “xi” represents the presence (1) or absence (0) of the risk factor, and “wi” represents its specific weight. Each factor is assigned a weight according to its relative risk (Risk Ratio) reported in (Livingston et al., 2020), resulting in a total score ranging from 0 to 40. The weights are as follows: low education (7), hearing loss (8), traumatic brain injury (3), hypertension (2), alcohol abuse (1), obesity (1), smoking (5), depression (4), social isolation (4), physical inactivity (2), air pollution (2), and diabetes (1).

#### 2.2.2 Clinical history (anamnesis) interview

Baseline socio-demographic and clinical characteristics were collected using an *ad hoc* structured interview questionnaire. The tool systematically records core demographic data (including education, age, gender), and a comprehensive medical history checklist covering self-reported medical, cognitive, and neuropsychiatric disorders, regular medication intake, and an examiner observation registry.

#### 2.2.3 Mini mental state examination

The MMSE (Folstein et al., 1975) is an orally administered and widely recognized tool used to evaluate cognitive function in individuals suspected of having cognitive impairments. It assesses several domains, including orientation to time and place, short-term memory, attention, and arithmetic skills. The test consists of a series of questions and tasks, with a maximum possible score of 30 points; lower scores suggest more severe cognitive deficits. In a revised normative study involving participants from Southern Italy (Carpinelli Mazzi et al., 2020), multiple linear regression analysis revealed that age and education significantly influenced the MMSE scores, while sex did not. A cut-off score of 24.9 out of 30 successfully identified 44 of 47 individuals diagnosed with Alzheimer’s Disease. It is the most widely used test for assessing cognitive functions in studies on cognitive interventions (training and rehabilitation) in individuals with dementia (Giaquinto et al., 2025). Administration modality used in this study are the same expressed in the mentioned validation studies.

#### 2.2.4 Montreal cognitive assessment

The MoCA (Nasreddine et al., 2005) is a screening tool designed to identify Mild Cognitive Impairment. It evaluates a range of cognitive domains, such as memory, language, executive functioning, visuospatial skills, calculation, abstraction, attention, concentration, and orientation. Similar to the MMSE, it includes a series of questions and tasks with a total possible score of 30, where lower scores reflect greater cognitive impairment. In the Italian normative study (Santangelo et al., 2015), multiple linear regression analysis indicated that age and educational level significantly affected MoCA scores, whereas sex did not. The inferential cut-off score was set at 15.5. A statistically significant, moderately positive correlation was observed between MoCA and MMSE-adjusted scores in cognitively healthy population conducted in Italy (e.g., Santangelo et al. (2015): *r* = 0.43; *p* < 0.001; Giaquinto et al. (2024): *r* = 0.31; *p* < 0.001). Administration modality used in this study are the same expressed in the mentioned validation studies.

#### 2.2.5 Self-administered tasks uncovering risk of neurodegeneration

The SATURN (Bissig et al., 2020) is a digital, self-administered tool designed to assess global cognitive functioning and detect cognitive decline, originally introduced in the USA (Giaquinto et al., 2022). Its Italian version was recently validated (Giaquinto et al., 2024). This version consists of 19 tasks, with a maximum total score of 29, and evaluates domains such as attention, incidental and recall memory, temporal orientation, mathematical skills, visuo-constructional abilities, and executive functions. The test provides both total and domain-specific scores for accuracy and response time. In this study, the SATURN was administered via laptop using a mouse. Participants completed the test independently; however, those with limited digital literacy received assistance from a researcher for input only, without support in reading or interpreting the tasks. According to Italian normative data, the non-parametric lower cut-off for total accuracy was set at 16.46 out of 29. In a cognitively healthy Italian sample, statistically significant moderate positive correlations were observed between SATURN and MMSE scores (*r* = 0.19; *p* < 0.001), as well as between SATURN and MoCA scores (*r* = 0.30; *p* < 0.001) (Giaquinto et al., 2024). Administration modality used in this study are the same expressed in the mentioned validation studies.

#### 2.2.6 Frontal assessment battery

The FAB [Frontal Assessment Battery, (Apollonio et al., 2005)] is a commonly used screening tool for detecting executive function deficits (Hurtado-Pomares et al., 2018). It evaluates several aspects of executive functioning, including conceptualization, cognitive flexibility, motor programming, interference control, inhibitory control, and autonomy in environmental interaction. Research conducted on the Italian population has reported sensitivity rates between 61.1 and 77.8%, and specificity rates ranging from 64.4 to 80.3%, for identifying general cognitive inefficiency associated with executive dysfunction (Aiello et al., 2022). Administration modality used in this study are the same expressed in the mentioned validation studies.

#### 2.2.7 Geriatric depression scale

The Geriatric Depression Scale [GDS, (Yesavage et al., 1982)], is a widely recognized tool for evaluating depressive symptoms in older adults. This study utilized the 15-item short form of the GDS, which assesses emotional and behavioural indicators of depression experienced over the previous week (Sheikh and Yesavage, 1986). Each item requires a binary response—“yes” or “not.” Typically, a “yes” response is scored as 1 and a “not” as 0; however, certain items are reverse-scored (i.e., “yes” = 0, “not” = 1) to mitigate response bias and enhance the accuracy of the assessment. The total score, calculated by summing the responses, ranges from 0 to 15, with higher scores indicating more pronounced depressive symptoms. The Italian version of the GDS used in this study has demonstrated strong psychometric qualities, including high reliability and validity for use with older Italian-speaking populations (Chiesi et al., 2017). Moreover, the GDS is the most frequently used tool in Italy’s Centers for Cognitive Disorders and Dementias (Bacigalupo et al., 2024; Rizzi et al., 2025) and is also the primary measure employed in research evaluating the effects of cognitive training in individuals with dementia (Giaquinto et al., 2025). Administration modality used in this study are the same expressed in the mentioned validation studies.

### 2.3 Procedure

The administration of the tests was conducted by psychologists with expertise in neuropsychology, with the collaboration of psychology students who were adequately trained and supervised throughout the phases of data collection, scoring, and tabulation. The testing took place in various settings depending on the recruitment method: at the general practitioner’s office, at the pharmacy, or at the participant’s home. In all cases, testing was conducted in person in a quiet room free from distractions, during daylight hours, with natural lighting, and in the presence of one or two researchers. When two researchers were present, the second acted as an observer and aided with the administration of digital tests in cases where the participant had limited computer skills. This digital assistance involved helping the participant enter responses using the mouse, based on their verbal instructions. Researchers were instructed not to read or interpret the questions or the responses during the digital test administration. This method was found not to affect accuracy scores or response times (Giaquinto et al., 2024). To minimize participant fatigue and guarantee optimal engagement, the total assessment duration was strictly limited to a maximum of 60 min. A sequential enrolment protocol was adopted, structured as follows: a core assessment battery comprising clinical history (anamnesis), SATURN (digital format), MoCA, RF12-Self (digital format), and GDS was administered to the entire sample. Conversely, due to the aforementioned time constraints, the MMSE and FAB were not administered to all participants; instead, they were randomly alternated across two distinct subsamples. This randomized allocation ensured that missingness was due to time management rather than clinical or demographic characteristics. Finally, the RF12-Informant version was administered only when a reliable informant (e.g., a family member or caregiver) was available to complete the questionnaire. The RF12 and SATURN tests were administered using PsychoPy software version 2022.2.2 (Peirce et al., 2019), with local data storage on a laptop and mouse input, at 70 centimetres from the monitor. Stimuli sizes were adjusted proportionally to the dimensions of the monitor used. All data were subsequently extracted, cross-checked, and tabulated using anonymization procedures in accordance with the approved ethical protocol. Within one week of the initial assessment, if possible, a cohabiting partner or family member of the participant was interviewed and asked to complete the RF12 – Informant version, via an online web survey accessible on a smartphone, tablet, or laptop. Data collection took place from April 2023 to October 2024.

### 2.4 Statistical analysis

The data analysis was conducted in multiple steps to examine relationships between modifiable risk factors and cognitive as well as affective outcomes. First, descriptive statistics were computed to summarize the demographic and clinical characteristics of the sample, including measures of central tendency and dispersion for age, education, and test scores. Spearman’s Rho correlation coefficients were calculated to explore associations between the RF12 indices and cognitive and depressive symptom measures, due to the non-parametric distribution of some variables. Subsequently, hierarchical regression analyses were performed to evaluate the incremental contribution of each RF12 scoring method (RF12-Self, RF12-Weighted, RF12-Informant) to the variance in cognitive and affective outcomes, while controlling for potential confounding variables such as age, sex, and education level. Predictor variables were entered sequentially in blocks to quantify the additional variance explained by each RF12 version. Statistical significance was set at *p* < 0.05 for all analyses. All statistical procedures were conducted using JASP software, version 0.93 (JASP Team, 2024), and JAMOVI, version 2.6 (Jamovi Project, 2024).

### 2.5 Ethical approval

The research involving human participants received approval from the National Ethics Committee for experiments conducted by public research institutions (NEC) and other national public bodies (CEN) during the session held on April 4, 2023 (Project Code: PNRR-MAD-2022-12376781). All procedures were carried out in compliance with applicable national regulations and institutional guidelines. Written informed consent was obtained from all participants prior to their involvement in the study.

## 3 Results

### 3.1 RF12 description

Table 1 presents descriptive statistics of RF12 and the other variables under investigation. The RF12 self-report and informant-report versions showed a significant positive correlation (Spearman’s *ρ* = 0.408, *p* < 0.001, df = 149). While statistically significant, the modest magnitude of this correlation suggests potential discrepancies between self- and informant-reported information, consistent with a self–other knowledge asymmetry (Vazire, 2010).

**Table 1:** Descriptive statistics of the RF12, the cognitive tests assessed, and the depression scale.

Variables	*N*	Median	Mean	Std. deviation	Min–Max
Age	563	61	61.79	10.19	27–91
Education	563	13	11.76	4.61	3–30
RF12_Self	512	2.0	2.15	1.47	0–8
RF12_S_W	512	9.0	9.15	6.55	0–32
RF12_info	174	3.0	3.36	2.74	0–6
MMSE	311	29.0	28.67	1.43	24–30
MoCA	563	24.0	24.16	3.48	13–30
SATURN	543	25.0	24.25	3.12	15–29
FAB	252	16.0	15.28	2.39	3–18
GDS	562	3.0	4.18	3.33	0–15

Age: expressed in years; Education: expressed in years of education; RF12_Self: RiskFactors-12 items—self-administered version; RF12_S_W: RiskFactors-12 items with weights calculation based on (Livingston et al., 2020); RF12_info: RiskFactors-12 items: informant version; MMSE: Mini Mental State Examination; MoCA: Montreal Cognitive Assessment; SATURN: Self-Administered Tasks Uncovering of Risk for Neurodegeneration; FAB: Frontal Assessment Battery; GDS: Geriatric Depression Scale.

Frequencies of individual modifiable risk factors are reported in Table 2. The most prevalent risk factors were low educational attainment (39.3%), smoking (34.4%), and insufficient aerobic physical activity (<60 min/week; 33.7%), whereas the least common were lifetime traumatic brain injury (5.1%), significant hearing loss >10 dB (6.3%), and alcohol abuse (7.4%).

**Table 2:** Frequencies of individual modifiable risk factors for dementia.

Modifiable risk factors	*N*	Frequency	Total positive
Low education (<9 yrs)	563	0.393	221
Smoking	511	0.344	176
Physical inactivity	511	0.337	172
High BMI (>30)	504	0.335	169
Hypertension	511	0.299	153
Poor social life	511	0.233	119
Air pollution	511	0.161	82
Diabetes	511	0.102	52
Alcohol abuse	511	0.074	38
Hearing problems	511	0.063	32
Depression	511	0.059	30
Traumatic brain injury	511	0.051	26

BMI: body mass index.

Table 3 shows associations between the different RF12 versions and cognitive performance measures. A weighted version of the RF12, in which each risk factor was adjusted according to weights derived from the Lancet Commission’s estimates of their relative contributions (Livingston et al., 2020), was also examined. Although this weighted version explained slightly more variance in some outcomes, the practical benefits of the added computational complexity require further evaluation (see next section).

**Table 3:** Correlations (Spearman’s Rho) between the RF indices and the cognitive test scores.

Variables	RF12-self	RF12-informant	RF12-weighted
MMSE	−0.131*	−0.172*	−0.221***
MoCA	−0.265***	−0.119	−0.337***
SATURN	−0.197***	−0.301***	−0.226***
FAB	−0.333***	NA	−0.389***
GDS	0.209***	0.025	0.178***

RF12-Self: Risk Factors-12 items, self-administered version; RF12-informant: Risk Factors-12 items, informant version; RF12-weighted: Risk Factors-12 items with weights scoring based on (Livingston et al., 2020); MMSE: Mini Mental State Examination; MoCA: Montreal Cognitive Assessment; SATURN: Self-Administered Tasks Uncovering Risk for Neurodegeneration; FAB: Frontal Assessment Battery; GDS: Geriatric Depression Scale; Correlation are expressed in terms of Spearman’s Rho, significance as *p < 0.05, ***p < 0.001, (degrees of freedom), NA indicates Non Available data.

Overall, higher RF12 scores—whether self-reported, informant-reported, or weighted—were associated with lower cognitive test scores and higher depressive symptom scores, although the proportion of variance explained was modest.

### 3.2 Incremental validity

We examined the extent to which each version of the RF index (self-report, informant-report, and weighted) contributed to explaining variance in cognitive and affective outcomes. This analysis aimed to investigate the degree of independence between self- and informant-based reports, and to determine whether the additional computational complexity introduces by the weighted score translates into measurable incremental contributions to the variance of outcome measures.

To this end, five hierarchical regression models were constructed and compared using adjusted R^2^ values. Significance tests of incremental contribution were also conducted to assess whether the inclusion of each successive RF12 version resulted in statistically meaningful improvements in model fit. The models were built sequentially, with each new model incorporating all predictors from the previous model plus one additional RF12 score version. This approach allowed us to quantify the unique contribution of each RF12 scoring method while controlling for socio-demographic factors such as age, sex, and education.

- M_1_: included education, age, and sex as socio-demographic characteristics.
- M_2_: added the informant-reported RF12 score.
- M_3_: introduced the self-reported RF12 score.
- M_4_: included the weighted RF12 score.

The informant perspective was entered before the self-report version, based on the rationale that individuals generally have more accurate access to their own health-related information. This allowed assessment of whether self-reports contribute additional variance beyond informant reports. Results are reported in Table 4.

**Table 4:** Hierarchical regression analysis for cognitive and affective outcomes.

Model	*R* ^2^	Adjusted *R*^2^	Δ*R*^2^	df1	df2	*p*
MOCA						
M₁	0.206	0.201				
M₂	0.211	0.205	0.005	1	501	0.068
M₃	0.234	0.226	0.023	1	500	<0.001
**M₄**	**0.246**	**0.237**	**0.012**	**1**	**499**	**0.005**
MMSE						
**M₁**	**0.083**	**0.073**				
M₂	0.083	0.069	<0.001	1	266	0.934
M₃	0.083	0.066	<0.001	1	265	0.879
M₄	0.086	0.065	0.003	1	264	0.396
SATURN						
M₁	0.195	0.190				
**M₂**	**0.204**	**0.197**	**0.008**	**1**	**484**	**0.024**
M₃	0.207	0.199	0.003	1	483	0.149
M₄	0.210	0.200	0.003	1	482	0.176
FAB						
M₁	0.175	0.164				
M₂	0.184	0.170	0.009	1	230	0.112
**M₃**	**0.199**	**0.181**	**0.015**	**1**	**229**	**0.040**
M₄	0.199	0.178	0.103	1	228	0.748
GDS						
M₁	0.048	0.042				
M₂	0.060	0.053	0.013	1	500	0.010
**M₃**	**0.078**	**0.068**	**0.012**	**1**	**499**	**0.002**
M₄	0.078	0.067	<0.001	1	498	0.531

M₁ includes: education + age + sex. M₂ includes: M₁ + RF12-Informant. M₃ includes: M_2_ + RF12-Self. M₄ includes: M_3_ + RF12-Weighted. RF12-Self: Risk Factors-12 items, self-administered version; RF12-informant: Risk Factors-12 items, informant version; RF12-weighted: RiskFactors-12 items with weights scoring based on (Livingston et al., 2020); MMSE: Mini Mental State Examination; MoCA: Montreal Cognitive Assessment; SATURN: Self-Administered Tasks Uncovering Risk for Neurodegeneration; FAB: Frontal Assessment Battery; GDS: Geriatric Depression Scale. Bold values indicate the statistically preferred or optimal model selected for each outcome measure. This represents the final hierarchical step that resulted in a statistically significant change in variance explained (ΔR2, *p* < 0.05), or the baseline model (M1) if no subsequent steps yielded a significant improvement.

The results of hierarchical regression analysis indicate that the RF12-self version provides a clearly identifiable and unique contribution to the variance in FAB and GDS scores. For these measures, which are widely used to assess global cognitive functioning and depressive symptoms, respectively, the number of self-reported risk factors (RF-12 self) explains additional variance beyond that accounted for by socio-demographic predictors (age, sex, and education), thereby improving overall model fit. To assess the robustness of these findings and rule out potential inflation due to circularity between the historical depression item of the RF12 and the current symptomatology of the GDS, a sensitivity analysis was performed using a modified 11-item index (RF11) that excludes the depression item. Omission of this item resulted in a minor decrease in the final explained variance (dropping from *R*^2^ = 0.078 to *R*^2^ = 0.062). Notably, while the step of maximum significance occurred at Model 3 in the primary RF12 analysis ( Δ *R*^2^ = 0.012, *p* = 0.040), it shifted to Model 2 in the sensitivity RF11 analysis ( Δ *R*^2^ = 0.011, *p* = 0.012), indicating that the depression item accounts for a small but unique portion of variance within the hierarchical steps. Regarding SATURN performance, the incremental contribution of the RF12 is primarily associated with the informant-reported version, whereas for MoCA scores, the weighted RF12 version accounts for additional variance, enhancing model fit. The only exception observed was the MMSE: once socio-demographic variables were included, adding any RF12 version did not appreciably improve model fit. This likely reflects the limited sensitivity of the MMSE in cognitively healthy populations rather than deficiencies in the RF12 indices themselves. Overall, these findings suggest that each RF12 scoring method contributes uniquely, although the proportion of variance explained remains modest across outcomes.

## 4 Discussion

This study examined the cross-sectional associations between RF12 scores and cognitive/affective measures, evaluated whether the RF12 explains additional variance beyond age, sex, and education, and assessed convergence between the self-report and informant-report versions. The rationale for introducing this tool stems from the growing recognition of lifestyle- and experience-related risk factors for dementia, which have implications for research, epidemiology, clinical practice, and personalized prevention strategies (Anstey et al., 2022). Additionally, the development considered the practical need for instruments suitable for primary care, where constraints such as limited time necessitate quick and interpretable first-level risk assessments (Sabbagh et al., 2020). However, at this stage, the RF12 should be understood as a low-burden, descriptive checklist of modifiable dementia-related risk factors rather than a diagnostic or prognostic instrument. Currently, no evidence-based cut-offs or clinical thresholds exist to distinguish “low” from “high” risk, meaning a provider cannot use specific scores (e.g., a score of 2 versus 8) to trigger automated clinical pathways or quantified risk estimates. Instead, in its present form, a higher score simply indicates a greater cumulative burden of potentially modifiable risk factors. In a primary care setting, this should serve as a practical prompt for clinicians to initiate patient discussions regarding prevention-oriented lifestyle changes and standard medical follow-ups for specific risk factors (e.g., cardiovascular health or hearing loss), rather than as a tool for formal risk stratification.

Work on integrated care policies has highlighted the important role of general practitioners in identifying early cognitive risk profiles (Bellomo et al., 2022). Therefore, it is necessary to develop practical tools that are applicable in such settings, easy to interpret, and quick to administer. The RF12 was designed as a concise instrument capturing the 12 risk factors identified by the 2020 Lancet Commission (Livingston et al., 2020) and updated in 2024 (Livingston et al., 2024). To firmly establish the clinical utility of the RF12, subsequent studies ought to conduct comprehensive validation assessments. Priorities for future research include examining test–retest reliability, assessing internal consistency where appropriate, and benchmarking the RF12 against existing dementia risk tools (e.g., LIBRA, CAIDE, ANU-ADRI) regarding the longitudinal prediction of cognitive decline and dementia outcomes.

An additional feature of the instrument is that it can be administered either as a self-report or informant-report. Involving an informant may be necessary when self-report is unreliable, for example due to cognitive, communication, or other health-related issues (Kroenke et al., 2022). In our community-dwelling sample, with self-reported absence of known neurological or psychiatric diagnoses, the self- and informant-reported RF12 scores were significantly correlated, though the moderate magnitude indicates a substantial self–other knowledge asymmetry. The moderate agreement observed between self- and informant-reported RF12 scores highlights a well-known challenge in gathering health-related data from multiple sources (e.g., Römhild et al., 2018). This discrepancy should not be automatically interpreted as evidence of the superiority of one perspective over the other. While patients typically have direct and accurate access to their personal medical history, cognitive or affective comorbidities might reduce their reliability. Conversely, informants might overlook less visible or early-stage risk factors (such as subtle hearing loss or subclinical mood changes) or may lack detailed knowledge of the patient’s remote history. This finding suggests that, at this stage, self- and informant-reports should be viewed as complementary sources of information rather than mutually exclusive or perfectly interchangeable measures. Determining which perspective, self or informant, is more reliable remains an open empirical question.

Hierarchical regression analyses showed that each RF12 version contributed uniquely to explaining variance in cognitive and affective outcomes. Specifically, RF12-Self explained additional variance in FAB and GDS scores beyond age, sex, and education; RF12-Informant contributed to SATURN performance; and RF12-Weighted explained additional variance in MoCA. MMSE scores did not show meaningful improvement, likely reflecting ceiling effects and limited sensitivity in cognitively healthy adults rather than limitations of the RF12 itself. This is consistent with the theoretical framework of the Scaffolding Theory of Aging and Cognition (Park and Reuter-Lorenz, 2009; Reuter-Lorenz and Park, 2014; Reuter-Lorenz and Park, 2024), which posits that lifestyle-related experiences can influence cognitive functioning with advancing age, both positively, e.g., (Giaquinto et al., 2023) and negatively. In our case, the RF-12 captures behavioral habits or events that may increase the risk of dementia either through their presence (e.g., being a smoker; having experienced traumatic brain injury) or their absence (e.g., insufficient physical activity; low educational attainment). Our findings show that, even in the general population, these risk factors influence cognitive functioning and the presence of cognitive symptoms as measured by psychometrically more sensitive tools for community samples. In particular, the RF-12 demonstrated a significant, but small, incremental contribution to the variance in global cognitive performance, as measured by the MoCA and SATURN, as well as in executive functioning, assessed via the FAB. However, no such effect was found for global cognition as measured by the MMSE. This lack of significance is likely due to the MMSE’s limited sensitivity in detecting subtle cognitive variations in individuals without manifest cognitive impairment, likely affected by ceiling effects and restriction of range (O’Bryant et al., 2010). However, it is crucial to note that the observed effect sizes (Δ*R*^2^ ranging between 0.8 and 2.3%) were small. While these findings provide preliminary evidence of an association between RF12 and current cognitive/affective measures at the group level, they do not imply that the instrument can currently guide individual clinical decisions. Establishing clinical meaningfulness, definitive cut-off scores, and decision thresholds will strictly require dedicated validation studies utilizing longitudinal outcomes. In its present form, the RF12 should be viewed as an exploratory index rather than a tool for individual risk stratification.

A potential methodological concern involves the shared construct variance between the history of depression included in the risk index and the active depressive symptoms measured by the GDS. However, our sensitivity analysis demonstrated that removing the historical item did not meaningfully alter the predictive value of the index. This suggests that the observed association reflects a well-documented clinical continuity between long-term vulnerability and current distress, rather than an artifact of methodological circularity, thereby supporting the conceptual validity of preserving the original structure of the index.

These results are consistent with findings from studies on other risk factor indices discussed in the literature, such as LIBRA, which have assessed the impact of lifestyle on cognitive performance and found a significant association between high-risk scores and lower cognitive performance in the general population (e.g., Wesenhagen et al., 2025). However, a study conducted on an Italian sample that included individuals with subjective cognitive decline and mild cognitive impairment (Franchini et al., 2019) found a significant correlation between LIBRA and cognitive performance in the former group but not in the latter. Another earlier study found that the assessment of risk factors predicts increased cognitive risk in midlife and late life, but not in the oldest-old (Vos et al., 2017). These findings suggest that the impact of lifestyle on cognitive performance may vary depending on an individual’s health status (Anatürk et al., 2023), highlighting the need for research across different sample types. In this perspective, a user-friendly tool such as the RF-12 could facilitate the conduction of large-scale epidemiological studies.

The informant version is a valuable option when the self-report cannot be considered reliable, though its predictive power appears to be less informative for FAB and GDS. Naturally, in the absence of a neurodegenerative disorder and when a reliable self-report is possible, the self-administered version should be preferred for data collection. However, in cases where the individual under clinical evaluation presents low reliability due to comorbid conditions, the informant version still shows a significant association with cognitive scores and with self-report scale. This dual-option availability is a valuable advantage for clinicians and increases the tool’s adaptability to different contexts of use.

Just like the index scores used in CAIDE, ANU-ADRI, and LIBRA, the RF-12 also includes a weighted scoring method, based on epidemiological evidence suggesting that different risk factors contribute unequally to the development of acquired neurodegenerative disorders and the risk ratios identified in (Livingston et al., 2020). In our sample, the weighted index demonstrated the strongest association with cognitive scores and accounted for additional explanatory power in MoCA performance among neurologically healthy, community-dwelling adults.

Associations between RF12 scores and depressive symptoms were also observed, as previously reported in a study involving a community-dwelling population aged 50 to 75 years using LIBRA (Geraets et al., 2023). Predicting depressive symptoms in midlife and late life does not equate to diagnosing depression. Depression has been linked to dementia in various studies, with some suggesting it may precede dementia as a prodromal symptom (Tetsuka, 2021) and others viewing it as an independent risk factor (Livingston et al., 2020), though the exact nature of the connection remains uncertain (Carrera-González et al., 2022). Therefore, it is crucial to pay close attention to depressive symptoms during the assessment phase for suspected cognitive decline. Modifiable risk factors are considered valuable targets for prevention of dementia risk associated with depressive symptoms, as even small reductions in risk could have a significant impact on public health at the population level (Asta et al., 2025).

The distribution of risk factors in the sample of the present study, randomly selected from the Apulia region in Southern Italy, generally reflects the regional distributions reported in a recent study conducted by the Italian National Institute of Health (Asta et al., 2025). There was a predominance of low educational attainment, alcohol consumption, and smoking rates above the national average. The distribution of risk factors can vary across different geographic and social contexts, influenced by differing lifestyles and life experiences across regions.

## 5 Limitations

This observation leads to several considerations regarding the limitations of the present study. First, the sample is not representative of the entire Italian population, as it reflects a specific region of Southern Italy (Apulia). Future studies aim to increase sample size and enhance representativeness. The ease of administration of the RF-12, combined with its concise format, supports the feasibility of large-scale studies. Furthermore, individuals with diagnosed neurological or psychiatric disorders were excluded, restricting the generalizability of findings. The impact of modifiable risk factors on cognitive performance may differ in clinical populations (O’Bryant et al., 2010). Future research should therefore extend the investigation to individuals with confirmed cognitive impairment to better understand the interplay of these variables. Second, the cross-sectional design precludes assessment of predictive validity. It remains unclear whether the RF-12 can forecast the likelihood of developing dementia, and longitudinal studies will be necessary to address this question. Longitudinal studies will be needed to test this hypothesis. Third, the current version of the tool includes only the 12 risk factors identified in the 2020 (Livingston et al., 2020). However, the 2024 update (Livingston et al., 2024) introduced two additional factors that are not yet included: hypercholesterolemia and vision loss. An item related to vision loss was already incorporated, as it was the subject of an ongoing study and was already suggested as a potential risk factor in 2020 (Livingston et al., 2020). Future updates should incorporate hypercholesterolemia to align the RF-12 with the latest evidence (Livingston et al., 2024). Another limitation of this study is the absence of a comparison between self-reported and objectively measured risk factors. For example, hearing loss was captured through self-report questionnaires rather than objective audiometric testing. Given that self-awareness of hearing decline can be limited in older populations, this reliance on self-report may have introduced underreporting bias (Tsimpida et al., 2020). To address this, future iterations of the tool should validate this item against objective measures or standardized subjective scales like the HSAQ (Bonetti et al., 2018). The binary scoring system inevitably reduces variability; however, it was chosen to maximize usability in contexts as primary care, where simplicity and speed are essential. An inherent limitation of the proposed weighted score lies in the assumption of additivity. Summing weights derived from epidemiological risk estimates assumes additivity and independence among risk factors, whereas the true associations may be multiplicative, non-linear, age-dependent, or moderated by interactions between risk factors. Accordingly, the weighted score should be considered a provisional index that requires external calibration and validation before being used for risk estimation or clinical interpretation. Research on modifiable risk factors has gained significant momentum in recent years, as the prevention of acquired neurodegenerative disorders currently remains the only viable path toward achieving major public health goals such as reducing global dementia cases. Consequently, practical tool for assessing risk factors must be designed for regular updates, to remain aligned with ongoing scientific advancements. Fourth, certain secondary measures were available only for specific subsamples. Due to strict time-management protocols aimed at avoiding cognitive fatigue, the MMSE and FAB were randomly alternated among participants, while the RF12-Informant score was inherently constrained by caregiver availability. Although the randomized allocation of the MMSE and FAB minimizes the risk of systematic selection bias, the smaller sample sizes for these specific instruments (*n* = 311 for MMSE, *n* = 252 for FAB, and *n* = 174 for RF12-Informant) inevitably result in reduced statistical power. Consequently, findings involving these sub-assessments should be interpreted with caution and require replication in larger, fully completed cohorts. Finally, the lack of validated clinical thresholds and explicit referral guidelines represents a current limitation that restricts the immediate translational utility of the RF12 in primary care. Future longitudinal and comparative studies are strictly required to establish evidence-based cut-offs, define distinct risk strata, and evaluate how the RF12 alters clinical decision-making compared to existing, more intensive risk assessments. Determining these thresholds will be essential to establish clear, actionable recommendations for providers based on specific score ranges.

## 6 Conclusion

In conclusion, the RF-12 appears to be a practical and efficient tool for assessing lifestyle- and experience-related modifiable risk factors for dementia. Each scoring method provides unique, albeit modest, contributions to explaining cognitive and affective outcomes. Findings are consistent with those from other composite indices, and the tool’s simplicity supports feasibility in research, epidemiological, and potentially clinical contexts. Further validation in diverse populations and longitudinal designs is warranted to establish broader utility and predictive relevance.

## Data Availability

The original contributions presented in the study are publicly available. This data can be found here: https://osf.io/k89ub/overview.
