# Supplementary material for: A brief checklist of modifiable dementia risk factors (RF12): associations with cognitive and affective measures in an Italian cohort
Source: Front Aging Neurosci. 2026 Jul 21;18:1862742. doi: 10.3389/fnagi.2026.1862742 (PMC13433509; doi:10.3389/fnagi.2026.1862742)
Supplement: Supplementary file 1 [file Table_1.docx]

Supplementary Material

**First-Level Assessment of Modifiable Risk Factors for Dementia: Introduction of the Risk Factors 12 items (RF12) Checklist**

| S.1. - Transparency Of Methods (TOM) (Zogmeister et al., 2024) |  | p. 2 |
| --- | --- | --- |
| S.2. - RF-12 - Self-administered version (Italian) |  | p. 5 |
| S.3. - RF-12 - Informant-administered version (Italian) |  | p. 6 |
| S.4. - RF-12 - Self-administered version (English) |  | p. 7 |
| S.5. - RF-12 - Informant-administered version (English) |  | p. 8 |
| S.6. - RF-12 – Modifiable Risk and Protective Factors for Dementia: Summary for the Proposed Checklist |  | p. 9 |

| **S.1. - Transparency Of Methods (TOM) (Zogmeister et al., 2024)*** | | | |
| --- | --- | --- | --- |
| **Item name** | **Description** | **Options** | **Response** |
| *variables.statement* | Is it stated that all measured variables and (if applicable) experimental conditions were reported?  For example:  "All measured variables are included in the analysis" = 1  "Variables X, Y, and Z were collected but they were used for a different study (reference / in preparation)" = 1  "Measured variables were A, B, C" with no further specification = 0 | 0 = There is no statement OR the statement is partial (e.g. concerns experimental conditions but not other variables)  1 = There are statements mentioning all study variables and, if applicable, all experimental conditions. | **1** |
| *open.materials* | Is it stated that materials (i.e. data OR additional analyses OR any other supplemental information) are openly available on a repository such as OSF, Zenodo, etc?  This information is typically reported before describing study methods or after the discussion (e.g., data availability statements) | 0 = There is no statement AND not all necessary materials were reported in the paper  1 = There is a statement about open materials OR there was no statement, but all necessary materials were reported in the paper  Insert a 9 if it is indicated that materials are available upon request | **Data: 9**  **New materials [RF12]: 1** |
| *population* | Are the characteristics of the population and all related inclusion or exclusion criteria reported in the paper?  For example,  "Participants were students." = 0  "Participants were university students from University X." = 1 | 0 = No  1 = Yes | **1** |
| *condition.assignment* | Is it clearly stated how participants were assigned to study condition (e.g., random assignment, within participants design)?  For example,  "As a between-subjects manipulation, half of the participants were assigned to perform a calibrated version of the estimation tasks. " = 0  "The block randomization option in the Qualtrics Survey Software randomly allocated participants into one of four conditions." = 1 | 0 = No  1 = Yes  Insert 9 if there was no assignment to study conditions (e.g. in correlational studies) | **9** |
| *demo.gender* | Are gender descriptives reported? | 0 = No  1 = Yes | **1** |
| *demo.age* | Are age descriptives reported? | 0 = No  1 = Yes | **1** |
| *demo.ethnicity* | Are ethnicity descriptives reported? | 0 = No  1 = Yes | **1** |
| *demo.educational* | Are education level descriptives reported?  For example:  if population comprises undergraduate students = 1. | 0 = No  1 = Yes | **1** |
| *demo.other* | Are other relevant demographic characteristics reported in the paper?  For example, medications, psychological conditions, diagnostic criteria.  NB: Here we refer only to those characteristics that might modulate the size of the effect(s) under investigation | 0 = there are other demographic characteristics relevant to the phenomena and they WERE NOT reported  1 = there are other demographic characteristics relevant to the phenomena and they WERE reported  Insert 9 if there are no other relevant demographic characteristics. | **1 (risk factors)** |
| *counterbalancing* | Is it clearly reported in the paper what was counterbalanced between participants (e.g., no counterbalancing, settings, tasks, stimuli)?  For example:  "half of the participants saw the congruent stimuli first, the other half saw the incongruent stimuli first" = 1  "tasks/stimuli were presented in the same fixed order to all participants" = 1  no specification = 0 | 0 = No  1 = Yes | **1** |
| *manipulation.check* | Is it clearly indicated whether and what manipulation check was used (e.g., no manipulation check, follow-up questions) ?  For example,  No mention to manipulation check in the paper and related materials = 0  If it is stated that there was no manipulation check = 1 | 0 = No  1 = Yes  Insert 9 if there were no experimental manipulations (i.e. correlational study) | **9** |
| *device* | Is it clearly indicated which devices were used to collect data (e.g., computer, tablet, smartphone, pen and paper)?  For example,  Online study: "Participants were asked to wear headphones, and automated procedures ensured that participants were all using the Google Chrome browser on a desktop computer." = 1  "Participants received the link to the form by email and completed the questionnaires on a computer, tablet or smartphone" = 1  Offline study: "The measures were taken on a computer using E-prime." = 1 | 0 = No  1 = Yes | **1** |
| *experimental.setting.detail* | Are the characteristics of the experimental setting reported?  NB: Here we refer only to those characteristics that, if manipulated, might modulate the size of the effect(s) under investigation (e.g., type of screen, room illumination for visual illusions, distance from the monitor, location of the experiment). | 0 = There are setting characteristics that might modulate the investigated effect(s) but they WERE NOT reported, or were only PARTIALLY reported.  1 = ALL setting characteristics that might modulate the investigated effect(s) WERE reported  Insert 9 if there are no setting characteristics that might modulate the size of the effect(s) under investigation. | **1** |
| *study.duration* | Is the duration of the study session(s) clearly indicated?  For example:  "the entire procedure lasted approximately one hour" = 1 | 0 = No  1 = Yes | **1** |
| *study.session.number* | Is it clear from the description of the procedure whether there were only one or several sessions?  For online studies, if it was not indicated whether participants were allowed to leave and re-enter the session = 0 | 0 = No  1 = Yes | **1** |
| *questionnaire.reference* | If the study includes questionnaires: is there a bibliographic reference for the questionnaire? | 0 = A published version of the questionnaire exists, but there is NO bibliographic reference to it.  1 = A published version of the questionnaire exists, and the paper includes a bibliographic reference.  Insert 9 if a reference for the questionnaire doesn't exist (e.g., it was newly developed) or the questionnaire is an adaptation or questionnaires were not used. | **1** |
| *grid.reference* | If the study includes observation grids (e.g. those used to assess child behaviour in a given situation): is there a bibliographic reference for the observation grid? | 0 = A published version of the grid exists, but there is NO bibliographic reference to it.  1 = A published version of the grid exists, and the paper includes a bibliographic reference.  Insert 9 if a bibliographic reference for the observation grid doesn't exist (e.g., it was newly developed) OR the observation grid was an adaptation or observational grids were not used. | **9** |
| * Zogmaister, C., Vezzoli, M., Facchin, A., Conte, F. P., Rizzi, E., Giaquinto, F., ... & Simioni, M. (2024). Assessing the Transparency of Methods in Scientific Reporting. *Collabra: Psychology*, 10(1). | | | |

| **S.2. - RF-12 - Self-administered version (Italian)** | | | | | |
| --- | --- | --- | --- | --- | --- |
| **#** | **Fattore di rischio** | **Domanda** | **Risposta** | **RF-12** | **RF-12-w** |
| 1 | BASSA SCOLARITÀ | Qual è il suo titolo di studi più elevato? |  |  |  |
| 2 |  | Fornisca la somma dei suoi anni di scolarità (ad esempio, se ha frequentato la scuola elementare per 5 anni, e le medie per 3 anni, i suoi anni di scolarità sono 5 + 3 = 8) |  | < 9: 1  $\geq$9: 0 | < 9: 7  $\geq$9: 0 |
| 3 | PERDITA DELL’UDITO | Soffre di una perdita dell’udito di almeno 10 dB? | Sì - No |  |  |
| 4 |  | Soffre di problemi di udito gravi tanto da avere difficoltà ad ascoltare una conversazione, ascoltare il telegiornale o chiede spesso di ripetere quanto si dice? (non indossa un apparecchio acustico e forse ne avrebbe bisogno) | Sì – No | Sì: 1  No: 0 | Sì: 8  No: 0 |
| 5 | TRAUMA CRANICO | Nel corso della vita ha mai subito frattura del cranio, edema, lezione cerebrale o emorragia (trauma cranico)? | Sì – No | Sì: 1  No: 0 | Sì: 3  No: 0 |
| 6 | IPERTENSIONE | Dai 30 anni in poi ha sofferto o soffre di ipertensione (pressione arteriosa minima o diastolica supera costantemente i 90 mm/Hg, mentre la massima o sistolica supera costantemente i 140 mm/Hg)? | Sì – No | Sì: 1  No: 0 | Sì: 2  No: 0 |
| 7 | ELEVATO CONSUMO DI ALCOL | Nel corso della sua vita ci sono mai stati periodi piuttosto prolungati in cui ha consumato bevande alcoliche (vino, birra, liquori, cocktail) almeno 3 volte al giorno per più di una settimana, o almeno 21 volte in una settimana? | Sì – No | Sì: 1  No: 0 | Sì: 1  No: 0 |
| 8 | OBESITÀ | Nel corso della sua vita, qual è il peso massimo che ha raggiunto? |  |  |  |
| 9 |  | Qual è la sua altezza (metri)? |  |  |  |
| 10 |  | BMI max = Kg/m^2^ = |  | >29.9 = 1 | >29.9 = 1 |
| 11 | FUMO | È un non fumatore da almeno 4 anni? | Sì – No | Sì: 0  No: 1 | Sì: 0  No: 5 |
| 12 | DEPRESSIONE | Ha sofferto di depressione clinicamente diagnosticata negli ultimi 10 anni? | Sì – No | Sì: 1  No: 0 | Sì: 4  No: 0 |
| 13 | VITA SOCIALE POVERA | Negli ultimi 5 anni, prende spesso parte ad attività sociali? | Sì – No | Sì-Sì: 0  Sì-No: 1  No-Sì: 1  No-No: 1 | Sì-Sì: 0  Sì-No: 4  No-Sì: 4  No-No: 4 |
| 14 |  | Ha qualcuno vicino su cui poter sempre contare? | Sì – No |  |  |
| 15 | ATTIVITÀ FISICA POVERA | Negli ultimi 10 anni, ha svolto attività fisica almeno una volta a settimana per 45-60 minuti (almeno camminata veloce)? | Sì – No | Sì: 0  No: 1 | Sì: 0  No: 2 |
| 16 | INQUINAMENTO ATMOSFERICO | Negli ultimi 10 anni, vive o ha vissuto in luoghi con un tasso di inquinamento sopra la media (area metropolitana, città con più di 80.000 abitanti, nei pressi di zone industriali, aeroporti, strade statali o autostrade, porti commerciali, …)? | Sì – No | Sì: 1  No: 0 | Sì: 2  No: 0 |
| 17 | DIABETE | Soffre di diabete (escluso diabete gestazionale transitorio)? | Sì – No | Sì: 1  No: 0 | Sì: 1  No: 0 |
| 18 | COVID-19 | Ha mai contratto il virus Covid-19? | Sì – No |  |  |
|  |  |  |  | ____/12 | ____/40 |

| **S.3. - RF-12 - Informant-administered version (Italian)** | | | | | |
| --- | --- | --- | --- | --- | --- |
| **#** | **Fattore di rischio** | **Domanda** | **Risposta** | **RF-12** | **RF-12-w** |
| 1 | BASSA SCOLARITÀ | Qual è il suo titolo di studi più elevato? |  |  |  |
| 2 |  | Fornisca la somma dei suoi anni di scolarità (ad esempio, se ha frequentato la scuola elementare per 5 anni, e le medie per 3 anni, i suoi anni di scolarità sono 5 + 3 = 8) |  | < 9: 1  $\geq$9: 0 | < 9: 7  $\geq$9: 0 |
| 3 | PERDITA DELL’UDITO | Soffre di una perdita dell’udito di almeno 10 dB? | Sì – No – Non so |  |  |
| 4 |  | Soffre di problemi di udito gravi tanto da avere difficoltà ad ascoltare una conversazione, ascoltare il telegiornale o chiede spesso di ripetere quanto si dice? (non indossa un apparecchio acustico e forse ne avrebbe bisogno) | Sì – No – Non so | Sì: 1  No: 0 | Sì: 8  No: 0 |
| 5 | TRAUMA CRANICO | Nel corso della vita ha mai subito frattura del cranio, edema, lezione cerebrale o emorragia (trauma cranico)? | Sì – No – Non so | Sì: 1  No: 0 | Sì: 3  No: 0 |
| 6 | IPERTENSIONE | Dai 30 anni in poi ha sofferto o soffre di ipertensione (pressione arteriosa minima o diastolica supera costantemente i 90 mm/Hg, mentre la massima o sistolica supera costantemente i 140 mm/Hg)? | Sì – No – Non so | Sì: 1  No: 0 | Sì: 2  No: 0 |
| 7 | ELEVATO CONSUMO DI ALCOL | Nel corso della sua vita ci sono mai stati periodi piuttosto prolungati in cui ha consumato bevande alcoliche (vino, birra, liquori, cocktail) almeno 3 volte al giorno per più di una settimana, o almeno 21 volte in una settimana? | Sì – No – Non so | Sì: 1  No: 0 | Sì: 1  No: 0 |
| 8 | OBESITÀ | Nel corso della sua vita, qual è il peso massimo che ha raggiunto? |  |  |  |
| 9 |  | Qual è la sua altezza (metri)? |  |  |  |
| 10 |  | BMI max = Kg/m^2^ = |  | >29.9 = 1 | >29.9 = 1 |
| 11 | FUMO | È un non fumatore da almeno 4 anni? | Sì – No – Non so | Sì: 0  No: 1 | Sì: 0  No: 5 |
| 12 | DEPRESSIONE | Ha sofferto di depressione clinicamente diagnosticata negli ultimi 10 anni? | Sì – No – Non so | Sì: 1  No: 0 | Sì: 4  No: 0 |
| 13 | VITA SOCIALE POVERA | Negli ultimi 5 anni, prende spesso parte ad attività sociali? | Sì – No – Non so | Sì-Sì: 0  Sì-No: 1  No-Sì: 1  No-No: 1 | Sì-Sì: 0  Sì-No: 4  No-Sì: 4  No-No: 4 |
| 14 |  | Ha qualcuno vicino su cui poter sempre contare? | Sì – No – Non so |  |  |
| 15 | ATTIVITÀ FISICA POVERA | Negli ultimi 10 anni, ha svolto attività fisica almeno una volta a settimana per 45-60 minuti (almeno camminata veloce)? | Sì – No – Non so | Sì: 0  No: 1 | Sì: 0  No: 2 |
| 16 | INQUINAMENTO ATMOSFERICO | Negli ultimi 10 anni, vive o ha vissuto in luoghi con un tasso di inquinamento sopra la media (area metropolitana, città con più di 80.000 abitanti, nei pressi di zone industriali, aeroporti, strade statali o autostrade, porti commerciali, …)? | Sì – No – Non so | Sì: 1  No: 0 | Sì: 2  No: 0 |
| 17 | DIABETE | Soffre di diabete (escluso diabete gestazionale transitorio)? | Sì – No – Non so | Sì: 1  No: 0 | Sì: 1  No: 0 |
| 18 | COVID-19 | Ha mai contratto il virus Covid-19? | Sì – No – Non so |  |  |
|  |  |  |  | ____/12 | ____/40 |

| **S.4. - RF-12 - Self-administered version (English)** | | | | | |
| --- | --- | --- | --- | --- | --- |
| **#** | **Risk factor** | **Question** | **Answer** | **RF-12** | **RF-12-w** |
| 1 | LOW EDUCATION | What is your highest level of education? |  |  |  |
| 2 |  | Please indicate the total number of years of schooling (e.g., 5 years of elementary school + 3 years of middle school = 8 years). |  | < 9: 1  $\geq$9: 0 | < 9: 7  $\geq$9: 0 |
| 3 | HEARING LOSS | Do you suffer from hearing loss of at least 10 dB? | Yes - No |  |  |
| 4 |  | Do you have serious hearing problems such that you struggle to follow conversations, watch the news, or frequently ask people to repeat themselves? (You do not wear a hearing aid but might need one.) | Yes - No | Yes: 1  Not: 0 | Yes: 8  Not: 0 |
| 5 | TRAUMATIC BRAIN INJURY | Have you ever experienced a skull fracture, swelling, brain injury, or haemorrhage (traumatic brain injury) in your lifetime? | Yes - No | Yes: 1  Not: 0 | Yes: 3  Not: 0 |
| 6 | HYPERTENSION | Since the age of 30, have you suffered from hypertension (diastolic pressure consistently above 90 mmHg, or systolic above 140 mmHg)? | Yes - No | Yes: 1  Not: 0 | Yes: 2  Not: 0 |
| 7 | HIGH ALCOHOL CONSUMPTION | At any point in your life, have there been extended periods during which you consumed alcoholic beverages (wine, beer, liquor, cocktails) at least 3 times per day for more than one week, or at least 21 times in one week? | Yes - No | Yes: 1  Not: 0 | Yes: 1  Not: 0 |
| 8 | OBESITY | What is the highest weight you have reached in your lifetime (in kg)? |  |  |  |
| 9 |  | What is your height (in meters)? |  |  |  |
| 10 |  | Max BMI = kg/m² = |  | >29.9 = 1 | >29.9 = 1 |
| 11 | SMOKING | Have you been a non-smoker for at least the past 4 years? | Yes - No | Yes: 0  Not: 1 | Yes: 0  Not: 5 |
| 12 | DEPRESSION | Have you been clinically diagnosed with depression in the past 10 years? | Yes - No | Yes: 1  Not: 0 | Yes: 4  Not: 0 |
| 13 | POOR SOCIAL LIFE | In the past 5 years, have you regularly taken part in social activities? | Yes - No | Yes-yes: 0  Yes-not: 1  Not-yes: 1  Not-not: 1 | Yes-yes: 0  Yes-not: 4  Not-yes: 4  Not-not: 4 |
| 14 |  | Do you have someone close you can always rely on? | Yes - No |  |  |
| 15 | POOR PHYSICAL ACTIVITY | In the past 10 years, have you engaged in physical activity at least once a week for 45–60 minutes (at least brisk walking)? | Yes - No | Yes: 0  Not: 1 | Yes: 0  Not: 2 |
| 16 | AIR POLLUTION | In the past 10 years, have you lived in areas with above-average air pollution (e.g., metropolitan areas, cities with more than 80,000 inhabitants, near industrial zones, airports, highways, or commercial ports)? | Yes - No | Yes: 1  Not: 0 | Yes: 2  Not: 0 |
| 17 | DIABETES | Do you suffer from diabetes (excluding temporary gestational diabetes)? | Yes - No | Yes: 1  Not: 0 | Yes: 1  Not: 0 |
| 18 | COVID-19 | Have you ever contracted COVID-19? | Yes - No |  |  |
|  |  |  |  | ____/12 | ____/40 |

| **S.5. - RF-12 - Informant-administered version (English)** | | | | | |
| --- | --- | --- | --- | --- | --- |
| **#** | **Fattore di rischio** | **Domanda** | **Risposta** | **RF-12** | **RF-12-w** |
| 1 | LOW EDUCATION | What is the highest level of education the person has completed? |  |  |  |
| 2 |  | Please indicate the total number of years of schooling the person has completed (e.g., 5 years of primary school + 3 years of middle school = 8 years). |  | < 9: 1  $\geq$9: 0 | < 9: 7  $\geq$9: 0 |
| 3 | HEARING LOSS | Does the person suffer from hearing loss of at least 10 dB? | Yes – No – I don’t know |  |  |
| 4 |  | Does the person have serious hearing problems such that they struggle to follow conversations, watch the news, or frequently ask others to repeat themselves? (They do not wear a hearing aid, but might need one.) | Yes – No – I don’t know | Yes: 1  Not: 0 | Yes: 8  Not: 0 |
| 5 | TRAUMATIC BRAIN INJURY | Has the person ever had a skull fracture, swelling, brain injury, or haemorrhage (traumatic brain injury)? | Yes – No – I don’t know | Yes: 1  Not: 0 | Yes: 3  Not: 0 |
| 6 | HYPERTENSION | Since the age of 30, has the person suffered from hypertension (diastolic >90 mmHg or systolic >140 mmHg consistently)? | Yes – No – I don’t know | Yes: 1  Not: 0 | Yes: 2  Not: 0 |
| 7 | HIGH ALCOHOL CONSUMPTION | Has the person ever had prolonged periods where they consumed alcoholic beverages (wine, beer, liquor, cocktails) at least 3 times a day for over a week, or at least 21 times in one week? | Yes – No – I don’t know | Yes: 1  Not: 0 | Yes: 1  Not: 0 |
| 8 | OBESITY | What is the highest weight the person has ever reached (in kg)? |  |  |  |
| 9 |  | What is the person's current height (in meters)? |  |  |  |
| 10 |  | Max BMI = kg/m² = |  | >29.9 = 1 | >29.9 = 1 |
| 11 | SMOKING | Has the person been a non-smoker for at least the past 4 years? | Yes – No – I don’t know | Yes: 0  Not: 1 | Yes: 0  Not: 5 |
| 12 | DEPRESSION | Has the person been clinically diagnosed with depression in the last 10 years? | Yes – No – I don’t know | Yes: 1  Not: 0 | Yes: 4  Not: 0 |
| 13 | POOR SOCIAL LIFE | In the past 5 years, has the person regularly participated in social activities? | Yes – No – I don’t know | Yes-yes: 0  Yes-not: 1  Not-yes: 1  Not-not: 1 | Yes-yes: 0  Yes-not: 4  Not-yes: 4  Not-not: 4 |
| 14 |  | Does the person have someone close they can always rely on? | Yes – No – I don’t know |  |  |
| 15 | POOR PHYSICAL ACTIVITY | In the last 10 years, has the person engaged in physical activity at least once a week for 45–60 minutes (at least brisk walking)? | Yes – No – I don’t know | Yes: 0  Not: 1 | Yes: 0  Not: 2 |
| 16 | AIR POLLUTION | In the past 10 years, has the person lived in areas with above-average pollution (metropolitan areas, cities with more than 80,000 inhabitants, near industrial zones, airports, highways, commercial ports, etc.)? | Yes – No – I don’t know | Yes: 1  Not: 0 | Yes: 2  Not: 0 |
| 17 | DIABETES | Does the person suffer from diabetes (excluding temporary gestational diabetes)? | Yes – No – I don’t know | Yes: 1  Not: 0 | Yes: 1  Not: 0 |
|  |  |  |  | ____/12 | ____/40 |

**S.6. - RF-12 – Modifiable Risk and Protective Factors for Dementia: Summary for the Proposed Checklist**

Evidence from the Lancet Commission (Livingston et al., 2020) and recent meta-analyses indicates that a significant proportion of dementia cases are linked to modifiable risk factors throughout the life course. The proposed checklist largely reflects these factors, utilizing operational thresholds adapted for clinical and screening purposes.

1 *Primary Risk Factors*

- **Low Educational Attainment:** “Low education” is identified as one of the key life-course factors with a high Population Attributable Fraction (PAF). Limited education in early life increases dementia risk and accounts for a substantial share of cases (Orgeta et al., 2018; Mukadam et al., 2019; Stephan et al., 2024; Jones et al., 2024; Beydoun et al., 2014; Chen & Kuper, 2023; Ma’u et al., 2021).
- **Hearing Loss:** Clinically significant hearing loss is a potent midlife risk factor, associated with an approximately twofold increase in risk and a high PAF; it is among the largest contributors to the global dementia burden (Orgeta et al., 2018; Livingston et al., 2025; Mukadam et al., 2019; Stephan et al., 2024; Jones et al., 2024; Senff et al., 2025; Rosenau et al., 2023; Chen & Kuper, 2023; Ma’u et al., 2021).
- **Traumatic Brain Injury (TBI):** Included among the 12 factors in the 2020 report, TBI is significantly associated with an elevated risk of dementia (Rolandi et al., 2020; Rosenau et al., 2023; Ma’u et al., 2021).
- **Hypertension:** Midlife hypertension is one of the most critical factors, characterized by a high PAF and robust empirical evidence (Orgeta et al., 2018; Mukadam et al., 2019; Stephan et al., 2024; Deckers et al., 2015; Beydoun et al., 2014; Rolandi et al., 2020; Walsh et al., 2022; Chen & Kuper, 2023; Li et al., 2020).
- **Obesity / BMI** $\geq$ **30:** Midlife obesity increases risk and maintains a consistent PAF across studies (Orgeta et al., 2018; Mukadam et al., 2019; Stephan et al., 2024; Deckers et al., 2015; Beydoun et al., 2014; Rolandi et al., 2020; Walsh et al., 2022; Chen & Kuper, 2023; Ma’u et al., 2021; Li et al., 2020).
- **Alcohol Consumption:** Excessive consumption was introduced as a new factor in 2020; evidence demonstrates an increased risk specifically associated with heavy usage (Deckers et al., 2015; Rosenau et al., 2023; Walsh et al., 2022; Chen & Kuper, 2023; Ma’u et al., 2021; Li et al., 2020).
- **Smoking:** Tobacco use is a confirmed risk factor; conversely, non-smoking status serves as a protective factor (Orgeta et al., 2018; Mukadam et al., 2019; Stephan et al., 2024; Jones et al., 2024; Deckers et al., 2015; Beydoun et al., 2014; Liang et al., 2020; Senff et al., 2025; Rosenau et al., 2023; Walsh et al., 2022; Chen & Kuper, 2023; Ma’u et al., 2021).
- **Clinical Depression:** Late-life depression is a major and well-documented risk factor (Orgeta et al., 2018; Stephan et al., 2024; Jones et al., 2024; Deckers et al., 2015; Beydoun et al., 2014; Rolandi et al., 2020; Rosenau et al., 2023; Chen & Kuper, 2023).
- **Diabetes:** Strongly associated with increased risk, supported by high-quality causal evidence (Orgeta et al., 2018; Mukadam et al., 2019; Stephan et al., 2024; Jones et al., 2024; Deckers et al., 2015; Beydoun et al., 2014; Liang et al., 2020; Rolandi et al., 2020; Walsh et al., 2022; Chen & Kuper, 2023; Ma’u et al., 2021; Li et al., 2020).
- **Air Pollution:** Added as one of the 12 primary factors; various analyses identify it as a significant emerging risk (Rosenau et al., 2023; Walsh et al., 2022; Chen & Kuper, 2023; Ma’u et al., 2021).

| **Checklist Item** | **Corresponding Epidemiological Factor** | **Citations** |
| --- | --- | --- |
| < 9 years of education | Low education | (Orgeta et al., 2018; Mukadam et al., 2019; Stephan et al., 2024; Jones et al., 2024; Beydoun et al., 2014; Rolandi et al., 2020; Chen & Kuper, 2023; Ma’u et al., 2021) |
| Hearing loss > 10 dB or severe | Hearing loss / sensory loss | (Orgeta et al., 2018; Livingston et al., 2025; Mukadam et al., 2019; Stephan et al., 2024; Jones et al., 2024; Senff et al., 2025; Rosenau et al., 2023; Chen & Kuper, 2023; Ma’u et al., 2021) |
| Hypertension for > 30 years | Midlife hypertension | (Orgeta et al., 2018; Mukadam et al., 2019; Stephan et al., 2024; Deckers et al., 2015; Rolandi et al., 2020; Chen & Kuper, 2023; Li et al., 2020) |
| BMI > 29 | Obesity (BMI $\geq30$) | (Orgeta et al., 2018; Mukadam et al., 2019; Stephan et al., 2024; Beydoun et al., 2014; Rolandi et al., 2020; Chen & Kuper, 2023; Ma’u et al., 2021; Li et al., 2020) |
| Binge drinking episodes | Excessive/heavy alcohol use | (Stephan et al., 2024; Jones et al., 2024; Deckers et al., 2015; Rosenau et al., 2023; Chen & Kuper, 2023; Ma’u et al., 2021; Li et al., 2020) |
| Diabetes | Diabetes mellitus | (Orgeta et al., 2018; Mukadam et al., 2019; Stephan et al., 2024; Jones et al., 2024; Deckers et al., 2015; Beydoun et al., 2014; Liang et al., 2020; Rolandi et al., 2020; Walsh et al., 2022; Chen & Kuper, 2023; Ma’u et al., 2021; Li et al., 2020) |
| Traumatic brain injury | Traumatic brain injury | (Jones et al., 2024; Rolandi et al., 2020; Rosenau et al., 2023; Chen & Kuper, 2023; Ma’u et al., 2021; Li et al., 2020) |

2 *Social and Lifestyle Protective Factors*

- **Regular Physical Activity:** Reduces risk, showing one of the highest PAFs among protective factors (Orgeta et al., 2018; Mukadam et al., 2019; Stephan et al., 2024; Jones et al., 2024; Deckers et al., 2015; Beydoun et al., 2014; Liang et al., 2020; Senff et al., 2025; Rosenau et al., 2023; Walsh et al., 2022; Chen & Kuper, 2023; Li et al., 2020).
- **Non-Smoking Status:** Remaining a non-smoker is protective compared to current smokers (Orgeta et al., 2018; Stephan et al., 2024; Jones et al., 2024; Deckers et al., 2015; Beydoun et al., 2014; Liang et al., 2020; Walsh et al., 2022; Chen & Kuper, 2023; Ma’u et al., 2021).
- **Social Engagement and Support:** Low social participation, isolation, or loneliness increases risk; conversely, active social participation and close interpersonal contacts are protective (Orgeta et al., 2018; Mukadam et al., 2019; Stephan et al., 2024; Jones et al., 2024; Peters et al., 2019; Senff et al., 2025; Rolandi et al., 2020; Rosenau et al., 2023; Chen & Kuper, 2023; Ma’u et al., 2021).

3 *Observations on Thresholds*

Existing literature utilizes standard clinical thresholds (e.g., BMI $\geq3$0, physical activity levels per WHO guidelines, clinical definitions of hypertension, hearing loss $\geq$20 dB, etc.) (Mukadam et al., 2019; Stephan et al., 2024; Rolandi et al., 2020; Chen & Kuper, 2023; Ma’u et al., 2021).

The thresholds established in the checklist (e.g., BMI > 29, 45–60 minutes of weekly activity, pragmatic definitions of severe hearing loss or heavy alcohol consumption) are thus anchored—per a precautionary approach—to these epidemiological cut-offs, translating empirical evidence into accessible screening criteria.

The proposed checklist is robustly supported: nearly every item corresponds to one of the 12 modifiable factors identified by the Lancet Commission or to factors confirmed by subsequent meta-analyses (traumatic brain injury, depression, social isolation, excessive alcohol consumption, obesity, physical inactivity, and diabetes). The specific thresholds (years of education, blood pressure values, BMI, alcohol frequency, or physical activity) represent pragmatic translations of the clinical and epidemiological definitions employed in international research.

**References**

Beydoun, M., Beydoun, H., Gamaldo, A., Teel, A., Zonderman, A., & Wang, Y. (2014). Epidemiologic studies of modifiable factors associated with cognition and dementia: systematic review and meta-analysis. *BMC Public Health, 14*, 643 - 643. <https://doi.org/10.1186/1471-2458-14-643>

Chen, S., & Kuper, H. (2023). Tracing the temporal trends of modifiable risk factors in dementia: insights from the English Longitudinal Study of Ageing (2004–2019). *The Lancet, 402*. <https://doi.org/10.1016/s0140-6736(23)02078-0>

Deckers, K., Boxtel, M., Schiepers, O., Vugt, M., Sánchez, J., Anstey, K., Brayne, C., Dartigues, J., Engedal, K., Kivipelto, M., Ritchie, K., Starr, J., Yaffe, K., Irving, K., Verhey, F., & Köhler, S. (2015). Target risk factors for dementia prevention: a systematic review and Delphi consensus study on the evidence from observational studies. *International Journal of Geriatric Psychiatry, 30*, 234 - 246. <https://doi.org/10.1002/gps.4245>

Jones, A., Ali, M., Kenny, M., Mayhew, A., Mokashi, V., He, H., Lin, S., Yavari, E., Paik, K., Subramanian, D., Dydynsky, R., Aryal, K., Correia, R., Dash, D., Manis, D., O'Connell, M., Liu-Ambrose, T., Taler, V., McMillan, J., Hogan, D., Kirkland, S., Costa, A., Wolfson, C., Raina, P., & Griffith, L. (2024). Potentially Modifiable Risk Factors for Dementia and Mild Cognitive Impairment: An Umbrella Review and Meta-Analysis. *Dementia and Geriatric Cognitive Disorders, 53*, 91 - 106. <https://doi.org/10.1159/000536643>

Li, X., Zhang, M., Xu, W., Li, J., Cao, X., Yu, J., & Tan, L. (2020). Midlife Modifiable Risk Factors for Dementia: A Systematic Review and Meta-analysis of 34 Prospective Cohort Studies.. *Current Alzheimer research*. <https://doi.org/10.2174/1567205017666200103111253>

Liang, J., Lu, L., Li, J., Qu, X., Li, J., Qian, S., Wang, Y., Jia, R., Wang, C., & Xu, Y. (2020). Contributions of Modifiable Risk Factors to Dementia Incidence: A Bayesian Network Analysis.. *Journal of the American Medical Directors Association*. <https://doi.org/10.1016/j.jamda.2020.04.006>

Livingston, G., Costafreda, S., Kivimäki, M., Mukadam, N., Schneider, L., Selbæk, G., Shirai, K., & Sommerlad, A. (2025). Reflections on The Lancet's Commission on dementia prevention, intervention, and care – Authors' reply. *The Lancet, 405*, 625-626. <https://doi.org/10.1016/s0140-6736(25)00150-3>

Ma’u, E., Cullum, S., Cheung, G., Livingston, G., & Mukadam, N. (2021). Differences in the potential for dementia prevention between major ethnic groups within one country: A cross sectional analysis of population attributable fraction of potentially modifiable risk factors in New Zealand. *The Lancet Regional Health: Western Pacific, 13*. <https://doi.org/10.1016/j.lanwpc.2021.100191>

Mukadam, N., Sommerlad, A., Huntley, J., & Livingston, G. (2019). DEMENTIA PREVENTION IN LOW- AND MIDDLE-INCOME COUNTRIES: POPULATION ATTRIBUTABLE FRACTION FOR POTENTIALLY MODIFIABLE RISK FACTORS. *Alzheimer's & Dementia, 15*. <https://doi.org/10.1016/j.jalz.2019.06.055>

Orgeta, V., Mukadam, N., Sommerlad, A., & Livingston, G. (2018). The Lancet Commission on Dementia Prevention, Intervention, and Care: a call for action. *Irish Journal of Psychological Medicine, 36*, 85 - 88. <https://doi.org/10.1017/ipm.2018.4>

Peters, R., Booth, A., Rockwood, K., Peters, J., D’Este, C., & Anstey, K. (2019). Combining modifiable risk factors and risk of dementia: a systematic review and meta-analysis. *BMJ Open, 9*. <https://doi.org/10.1136/bmjopen-2018-022846>

Rolandi, E., Zaccaria, D., Vaccaro, R., Abbondanza, S., Pettinato, L., Davin, A., & Guaita, A. (2020). Estimating the potential for dementia prevention through modifiable risk factors elimination in the real-world setting: a population-based study. *Alzheimer's Research & Therapy, 12*. <https://doi.org/10.1186/s13195-020-00661-y>

Rosenau, C., Köhler, S., Soons, L., Anstey, K., Brayne, C., Brodaty, H., Engedal, K., Farina, F., Ganguli, M., Livingston, G., Lyketsos, C., Mangialasche, F., Middleton, L., Rikkert, M., Peters, R., Sachdev, P., Scarmeas, N., Salbæk, G., Van Boxtel, M., & Deckers, K. (2023). Umbrella review and Delphi study on modifiable factors for dementia risk reduction. *Alzheimer's & Dementia, 20*, 2223 - 2239. <https://doi.org/10.1002/alz.13577>

Senff, J., Tack, R., Mallick, A., Gutiérrez-Martínez, L., Duskin, J., Kimball, T., Tan, B., Chemali, Z., Newhouse, A., Kourkoulis, C., Rivier, C., Falcone, G., Sheth, K., Lazar, R., Ibrahim, S., Pikula, A., Tanzi, R., Fricchione, G., Brouwers, H., Rinkel, G., Yechoor, N., Rosand, J., Anderson, C., & Singh, S. (2025). Modifiable risk factors for stroke, dementia and late-life depression: a systematic review and DALY-weighted risk factors for a composite outcome. *Journal of Neurology, Neurosurgery, and Psychiatry, 96*, 515 - 527. <https://doi.org/10.1136/jnnp-2024-334925>

Stephan, B., Cochrane, L., Kafadar, A., Brain, J., Burton, E., Myers, B., Brayne, C., Naheed, A., Anstey, K., Ashor, A., & Siervo, M. (2024). Population attributable fractions of modifiable risk factors for dementia: a systematic review and meta-analysis. *The Lancet. Healthy Longevity, 5*, e406 - e421. <https://doi.org/10.1016/s2666-7568(24)00061-8>

Walsh, S., Brain, J., Mukadam, N., Anderson, R., Greene, L., Govia, I., Kuhn, I., Anstey, K., Knapp, M., Stephan, B., & Brayne, C. (2022). A systematic review of the cost-effectiveness of community and population interventions to reduce the modifiable risk factors for dementia.. *Maturitas, 166*, 104-116. <https://doi.org/10.1016/j.maturitas.2022.09.002>
